# Anodal transcranial direct current stimulation does not alter GABA concentration or functional connectivity in the normal visual cortex

**DOI:** 10.3389/fnins.2025.1639838

**Published:** 2025-10-15

**Authors:** Dania Abuleil, Diana Gorbet, Daphne L. McCulloch, Remy Cohan, Jennifer Evelyn Kate Steeves, Ji Won Bang, Kevin C. Chan, Benjamin Thompson

**Affiliations:** ^1^School of Optometry and Vision Science, University of Waterloo, Waterloo, ON, Canada; ^2^Centre for Vision Research, York University, Toronto, ON, Canada; ^3^Spencer Center for Vision Research, Byers Eye Institute, Stanford University School of Medicine, Palo Alto, CA, United States; ^4^Centre for Eye and Vision Research, 17W Science Park, Hong Kong, China

**Keywords:** MRS, tDCS, binocular rivalry, GABA, Glx

## Abstract

**Introduction:**

Anodal direct current stimulation (a-tDCS) of the visual cortex is a potential rehabilitation tool for vision disorders such as amblyopia and macular degeneration. However, the underlying neural mechanisms are currently unknown. When applied to the human motor cortex, a-tDCS reduces the concentration of gamma-aminobutyric acid (GABA), an inhibitory neurotransmitter that modulates neuroplasticity. Our primary aim was to assess whether the same a-tDCS paradigm alters local GABA concentration when applied to the healthy primary visual cortex. We also measured the effect of a-tDCS on visual cortex resting-state connectivity and sought to replicate reported observations of an association between visual cortex GABA concentration and the dynamics of binocular rivalry.

**Methods:**

Fourteen participants with normal vision completed two brain imaging sessions at least 48 hours apart. In each session, binocular rivalry dynamics, primary visual cortex GABA and glutamate-glutamine (Glx) concentrations (via magnetic resonance spectroscopy (MRS)) and resting-state functional connectivity (via task-free fMRI) were measured at baseline. Real or sham a-tDCS (20 min, 2mA) was then applied to the visual cortex in a randomized sequence followed by a second set of MRS and fMRI measurements.

**Results:**

No between-session effects of a-tDCS on GABA or Glx concentration or resting-state functional connectivity were observed. A pre-planned within-session analysis revealed a significant increase in Glx following a-tDCS that did not withstand multiple comparisons correction. No consistent relationships between binocular rivalry dynamics and GABA concentration were apparent.

**Discussion:**

Together, our results suggest that a-tDCS effects on the visual cortex may differ from the GABA-associated mechanism in motor cortex.

## Introduction

Anodal transcranial direct current stimulation (a-tDCS) is a non-invasive brain stimulation technique that involves delivery of a direct electrical current to targeted brain regions using head-mounted electrodes (Nitsche and Paulus, 2000). When applied to the visual cortex of individuals with normal vision, a-tDCS can improve contrast sensitivity (Kraft et al., 2010) and vernier acuity (Reinhart et al., 2016), reduce crowding in peripheral vision (Chen et al., 2021; Raveendran et al., 2020), and increase the amplitude of visually evoked potentials (Antal et al., 2004; Bello et al., 2023; Kim et al., 2019). Enhanced visual perceptual learning has also been observed when training is combined with a-tDCS in typical individuals (Pirulli et al., 2013) and in individuals with amblyopia (Spiegel et al., 2013b). The potential of a-tDCS as a vision rehabilitation tool has also been explored (Park and Thompson, 2024). A single session of visual cortex a-tDCS improves contrast sensitivity in adults with amblyopia (Ding Z, et al., 2016), an effect that is accompanied by increased visual cortex excitability (Ding Z, et al., 2016) and by equalization of the cortical response to inputs from the amblyopic and fellow eyes (Spiegel et al., 2013a). Comparable results have been found using animal models of amblyopia (Castaño-Castaño et al., 2017; Castaño-Castaño et al., 2019a; Castaño-Castaño et al., 2019b). Preliminary evidence indicates that a single session of visual cortex a-tDCS can reduce lateral inhibition (a phenomenon linked to crowding) at the preferred retinal locus in patients with macular degeneration (Raveendran et al., 2021), and can differentially affect reading of English and Chinese characters in individuals with macular degeneration (Silva et al., 2022). Single session a-tDCS also improves visual field sensitivity and increases visually evoked potential amplitude in patients with glaucoma (Mei et al., 2024). Although the behavioral effects of visual cortex a-tDCS are well documented, the underlying neural mechanisms are not well understood. This contrasts with the use of a-tDCS to modulate motor cortex function. Reduced concentration of the inhibitory neurotransmitter gamma-aminobutyric acid (GABA) has been identified as a key mechanism through which motor cortex a-tDCS enhances cortical excitability and neuroplasticity (Bachtiar et al., 2015; Kim et al., 2014; Patel et al., 2017, 2019). It is conceivable that a-tDCS exerts a similar effect when applied to the visual cortex. In animal studies, there is evidence that anodal and cathodal tDCS of the visual cortex alters cortical excitability measured using visually evoked potentials (VEPs), an effect that is linked to modulation of GABA and glutamate concentrations. In a recent study in cats, cathodal tDCS resulted in a decreased VEP amplitude in, interpreted as reduced neural activity related to changes in glutamate concentration (Zhao et al., 2020). Anodal tDCS had the opposite effect. A recent systemic review and meta-analysis by Bello et al. (2023) reported a significant effect of transcranial electrical stimulation on VEP amplitudes in healthy adult humans whereby anodal tDCS (Ding F, et al., 2016; Dong et al., 2020; Lau et al., 2021) or transcranial alternating current stimulation (tACS; Nakazono et al., 2020) enhanced VEP amplitude.

GABA is an important modulator of visual cortex neuroplasticity (Sale, 2010; Stryker and Löwel, 2018) and changes in GABA concentration could explain vision improvements in patient populations following visual cortex a-tDCS (Thompson, 2021). Binocular rivalry dynamics may serve as an indirect measure of visual cortex GABA concentration. Binocular rivalry occurs when conflicting images are presented to each eye. The two images compete for perceptual dominance resulting in periods of complete suppression of one eye or the other (dominance) interspersed with periods of regional dominance of each eye (piecemeal) and fused (mixed) percepts. Higher concentrations of visual cortex GABA concentration have been associated with longer periods of dominance and pharmacological manipulations that increase GABA inhibition increase dominance durations (Mentch et al., 2019; Pitchaimuthu et al., 2017; van Loon et al., 2013). Therefore, if visual cortex a-tDCS reduces GABA concentration, shorter periods of dominance during binocular rivalry would be expected after stimulation. However, we found no effect of a-tDCS on binocular rivalry dynamics (Abuleil et al., 2021). This may have been because visual cortex a-tDCS does not influence GABA concentration or because binocular rivalry dynamics do not provide a reliable measure of visual cortex GABA concentration. Prior studies did not measure GABA directly or compare GABA concentrations with binocular rivalry dynamics following a-tDCS.

In this study, we investigated the mechanisms of visual cortex a-tDCS by directly measuring both GABA and glutamate-glutamine complex (Glx; an excitatory neurotransmitter) concentrations before and after stimulation using magnetic resonance spectroscopy. We also measured binocular rivalry dynamics at baseline to explore the reported association between visual cortex GABA concentration and rivalry dominance durations. Previous studies show that a-tDCS to motor areas can alter resting-state functional connectivity despite opposing effects (increased vs. decreased connectivity) across studies (Antonenko et al., 2017; Bachtiar et al., 2015). Based on these findings, resting-state functional connectivity was measured to assess whether visual cortex a-tDCS influences cortical networks. Our primary hypothesis was that real, but not sham, a-tDCS of the visual cortex would reduce visual cortex GABA concentration. Our secondary hypotheses were that binocular rivalry dominance durations would be positively associated with baseline visual cortex GABA concentration, and that real, but not sham, visual cortex a-tDCS would modulate resting-state functional connectivity within the visual cortex.

## Materials and methods

### Participants

Fourteen participants (mean age 27 years; range 20–39, 8 female) with normal or corrected-to-normal visual acuity (< 0.1 logMAR) and stereoacuity (≤ 40 arc sec on The Fly Stereo Acuity Test®, Vision Assessment Corporation) took part in the study. Participants were screened for MRI and brain stimulation safety and eligibility. Exclusion criteria included neurological conditions and use of psychoactive drugs. Participants were instructed to avoid alcohol the day before the study and caffeine the day of the study. All participants provided written informed consent. The study was approved by the University of Waterloo and York University Offices of Research Ethics and conformed to the principles of the Declaration of Helsinki. The sample size was chosen based on previous studies of tDCS effects on motor cortex GABA concentration (Stagg et al., 2009, *n* = 16; Allen et al., 2014, *n* = 16).

### Study design

**Figure 1:**
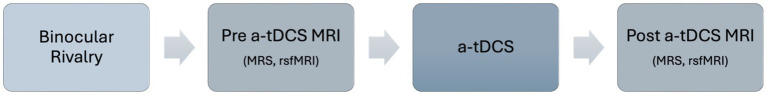
Experimental protocol. Participants took part in one active and one sham a-tDCS visits that were separated by at least 48 h. A-tDCS, anodal transcranial direct current stimulation; MRS, magnetic resonance spectroscopy; rsfMRI, resting-state functional magnetic resonance imaging.

Following a within-subjects study design, participants completed two visits: one for active stimulation and one for sham stimulation. The order of visits was randomized. Each visit consisted of a binocular rivalry psychophysical task, a 40-min MRS and fMRI scan, 20-min of visual cortex a-tDCS stimulation (active or sham), and finally another 40-min MRS and fMRI scan (Figure 1). Rivalry data were collected first outside the MRI room. Participants were then prepared for the MRI and given an eye mask to cover their eyes and given approximately 6 min to dark adapt while the scanner was prepared. No behavioral task was included during the MRI due to technical limitations in our scanning environment, as well as attempting to replicate previous studies which did not include a behavioral task (Kim et al., 2014). Following the baseline scan, participants were guided to the room outside the scanner where a-tDCS was performed while participants kept their eyes closed. Finally, participants were guided back to the MRI suite for post stimulation scanning. Visits were a minimum of 48 h apart to ensure any lasting effects of stimulation were diminished.

### Binocular rivalry

A binocular rivalry task was performed at the start of each visit using a CRT monitor to display two orthogonally oriented (45/135°) black and white gratings with a spatial frequency of 0.5 cycles per degree (cpd) within a circular field subtending 6.1° of visual angle on a mean luminance matched gray background as previously reported (Abuleil et al., 2020). Participants viewed the gratings dichoptically through a mirror stereoscope while sitting 75 cm away from the monitor. Participants reported their perception (45°, 135°, or piecemeal/mixed) using a keyboard. The total duration of each percept and the rate of alternation from one percept to another were analyzed.

### Transcranial direct current stimulation

Active anodal transcranial direct current stimulation (a-tDCS) and sham stimulation were delivered using a NeuroConn DC-Stimulator MC-8. The 5×7 cm electrodes were covered in a saline-soaked sponge and secured on the head with a head mount. The International 10–20 system was used for electrode placement. Both conditions (active and sham) involved placing the anodal electrode over Oz (approximately 2 cm above the inion) and the cathodal electrode over Cz. In the active condition, participants received 20 min of 2 mA stimulation in addition to a 30-s ramp up and 30-s ramp down period (Chen et al., 2021; Raveendran et al., 2020; Rushmore et al., 2013). The sham condition consisted only of the 30-s ramp up and 30-s ramp down periods.

### MRI acquisition

A 3-Tesla Siemens Magnetom® Tim Trio magnetic resonance scanner equipped with a 32-channel high-resolution brain array coil was used to acquire anatomical, spectroscopic, and resting-state fMRI data. Soft padding was placed around the participant’s head to minimize movement. Imaging data were acquired at rest and participants were instructed to keep their eyes closed throughout the scans. The sequence of spectroscopy and resting-state scans was counter-balanced across participants.

First, a three-dimensional T1-weighted magnetization prepared rapid gradient echo imaging (MPRAGE) sequence was used to acquire an anatomical volume [192 × 1.0 mm sagittal slices; in-plane resolution = 1 mm^2^; repetition time (TR)/echo time (TE) = 2300/2.26 ms; flip angle = 8°; field-of-view (FoV) = 256 mm; acquisition time = 5.21 min].

For proton (^1^H) magnetic resonance spectroscopy (MRS), a 2.5 × 2.5 × 2.5 cm voxel-of- interest (VOI) was placed over the primary visual cortex (V1). The VOI was centered on the calcarine sulcus and positioned to avoid non-brain tissue such as cerebrospinal fluid and the sagittal sinus. The Mescher-Garwood point-resolved spectroscopy (MEGA-PRESS) technique (Mescher et al., 1998) was used to record ^1^H MR GABA-edited spectra (TR/TE = 3000/68 ms; spectral bandwidth = 1,500 Hz; 2048 data points with water suppression yielding 32 averages; acquisition time = ~3:37). The acquisition was repeated 4 times for a total of 128 averages. Siemens standard and manual shimming were performed prior to each acquisition (details found in Stoby et al., 2022). The acquisition of ON and OFF edited spectra results in peaks affected by the editing pulses with GABA at approximately 3.02 ppm and Glx (glutamate and glutamine) at 3.80 ppm. This allows for the separation of GABA from creatine (Cr), an amino acid with a peak at 3.0 ppm. A water reference was acquired (1 average; acquisition time = 30 s).

Resting-state fMRI data were acquired using whole-brain multi-echo echo-planar imaging with a T2*-weighted sequence (43 contiguous axial slices; in-plane resolution = 3.4 × 3.4 mm^2^; slice thickness = 3.0 mm; imaging matrix = 64; TR = 3,000 ms; TE = 14.00 ms, 30.08 ms, 46.16 ms; flip angle = 83°; FoV = 216 mm; volumes = 205; acquisition time = 10 min 15 s).

### MRI analysis

*Magnetic Resonance Spectroscopy:* The Matlab based tool Gannet version 3.0 was used for analysis (Edden et al., 2014). Standard processing was performed for each acquisition, including frequency and phase correction, fast Fourier transformation and Gaussian model fitting of the GABA and Glx peaks to improve SNR and filter spectra. The total concentrations of GABA and Glx were estimated as the areas under the curve for GABA and Glx calculated using the GannetFit function. The GannetCoRegister function was used to register the chosen VOI to the anatomical image, using the program SPM8 (Statistical Parametric Mapping, Wellcome Centre for Human Neuroimaging, London, UK).^1^ GannetSegment performed segmentation of the anatomical images, and determined the relative amounts of gray matter, white matter, and CSF within the voxel, which allowed for the estimation of a CSF-corrected GABA and Glx concentration using SPM8. Lastly, GannetQuantify provided a tissue-corrected (relaxation- and alpha-corrected, voxel-average normalized) estimate of GABA and Glx concentrations. All concentrations were provided in institutional units (i.u.) relative to water. The standard deviation of the residual is used to determine the fit error of the model for each spectrum. All fit errors were <10%.

*Resting-State Functional Connectivity:* Resting-state functional MRI data were run through a series of preprocessing steps to minimize noise using multi-echo independent component analysis (ME-ICA) (Kundu et al., 2013) in Analysis of Functional Neuro Images toolbox (AFNI).^2^ A high-pass filter was applied to remove temporal frequencies less than 3 cycles/run, as well as a linear trend filter to remove scanner-related signal drift. Acquisition details can be found in our previous publication (Stoby et al., 2022). One participant’s data were discarded as they could not be processed reliably for technical reasons including excessive movement during scans.

**Figure 2:**
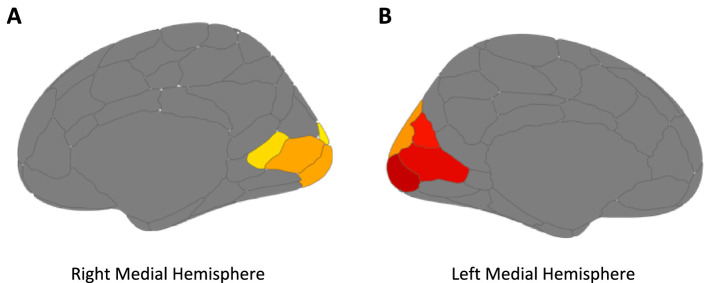
Brain regions chosen for voxel analysis of resting-state functional connectivity using Schaefer Atlas parcellation (Schaefer et al., 2018) in the right **(A)** and left; **(B)** medial hemisphere along the calcarine fissure. Colors are arbitrary.

MRI data were automatically parcellated into functional networks for each individual subject using Group Prior Individual Parcellation (GPIP) analysis (Chong et al., 2017). First, T1 images were parcellated using the recon-all function in Freesurfer (v6.0.1).^3^ Functional data were then transformed to the surface space using trilinear volume-to-surface interpolation (mri_vol2surf). Matlab was used to normalize each resting-state imaging run to a mean of 0 and a standard deviation of 1. Within GPIP, the data were initialized using the 200-parcel 7-Network Schaefer atlas (Schaefer et al., 2018; Figure 2). The program then optimized the parcel boundary using each subject’s resting-state functional images which were then visually inspected. Twenty iterations were performed through GPIP with increasingly refined resting-state network parcellations and the quality of GPIP parcellations was assessed by calculating homogeneity for each iteration. Homogeneity was calculated as the mean correlation coefficient of all pairs of vertices within each parcel averaged over all parcels in the brain for each subject to verify that these values increased over iterations and then plateaued in value prior to the final GPIP iteration. The mean time-series was calculated for each GPIP parcel included in the analysis. Pairwise Fisher Z values were calculated for all parcels included in the analysis.

### Statistical analysis

The effect of a-tDCS on GABA concentration was assessed using a repeated measures ANOVA with factors of Condition (active vs. sham) and Time (pre vs. post). Pre-planned paired-sample t-tests were performed to assess the pre-post differences in the active and sham conditions separately. The same analysis was conducted on the Glx concentration data to test for a-tDCS effects. Pearson correlation coefficients were used to assess the relationships among binocular rivalry dynamics (alternation rate and mixed percept duration) and concentrations of GABA and Glx at baseline. The effect of a-tDCS on functional connectivity was assessed using repeated measures ANOVAs with factors of Condition and Time performed on the Fisher Z values on the average of V1 visual network connectivity along the calcarine fissure.

## Results

**Figure 3:**
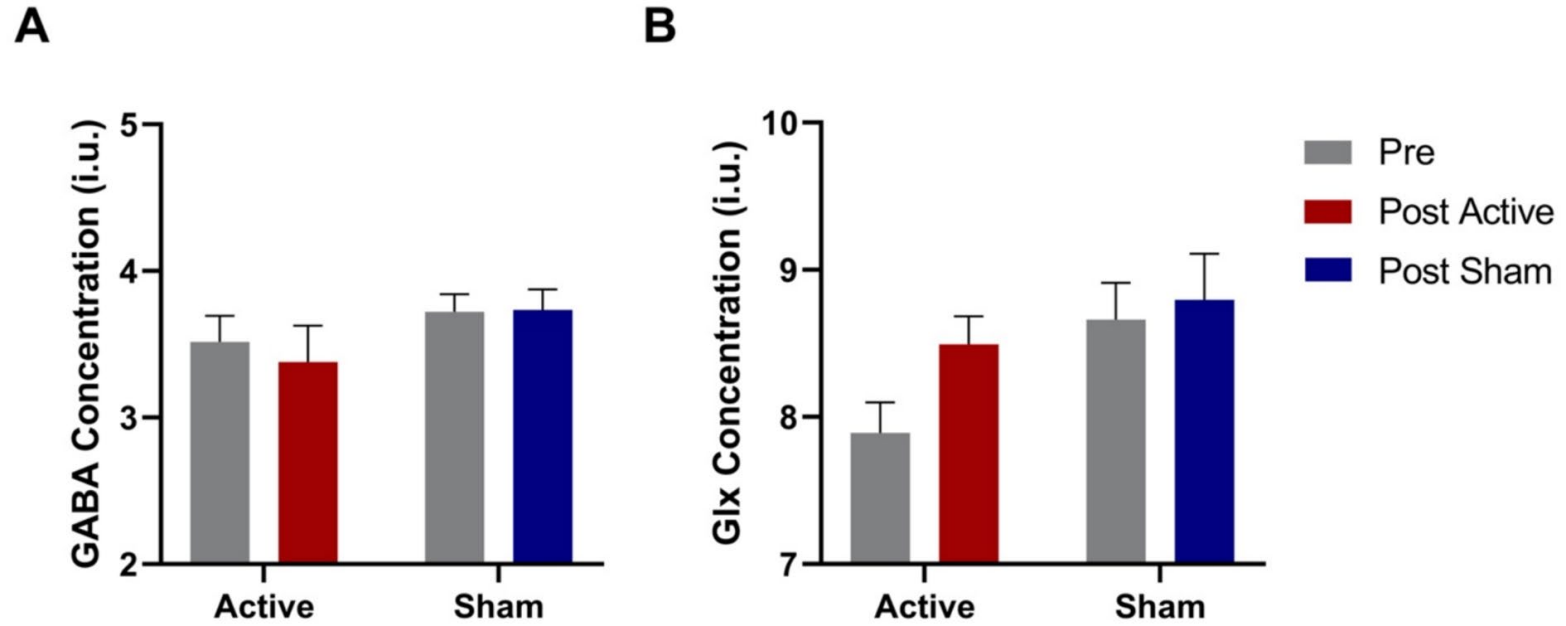
Pre and post GABA **(A)** and Glx **(B)** concentrations in the visual cortex for both active and sham conditions. *N* = 13. Error bars = SEM.

The results of mean visual cortex GABA and Glx concentrations pre- and post- real or sham a-tDCS are shown in Supplementary Table 1. There was no interaction between Condition and Time for either GABA or Glx (Figure 3), although GLx concentration was higher for the sham Condition (main effect, F_12_ = 5.732, *p* = 0.034). Pre-planned paired sample t-tests revealed a significant increase in Glx following active a-tDCS (t_12_ = 2.24, *p* = 0.045) that did not withstand multiple comparisons correction. No other pairwise comparisons was significant.

There was no interaction between Condition and Time for Fischer Z values of functional connectivity for the visual networks (*p* = 0.275).

**Figure 4:**
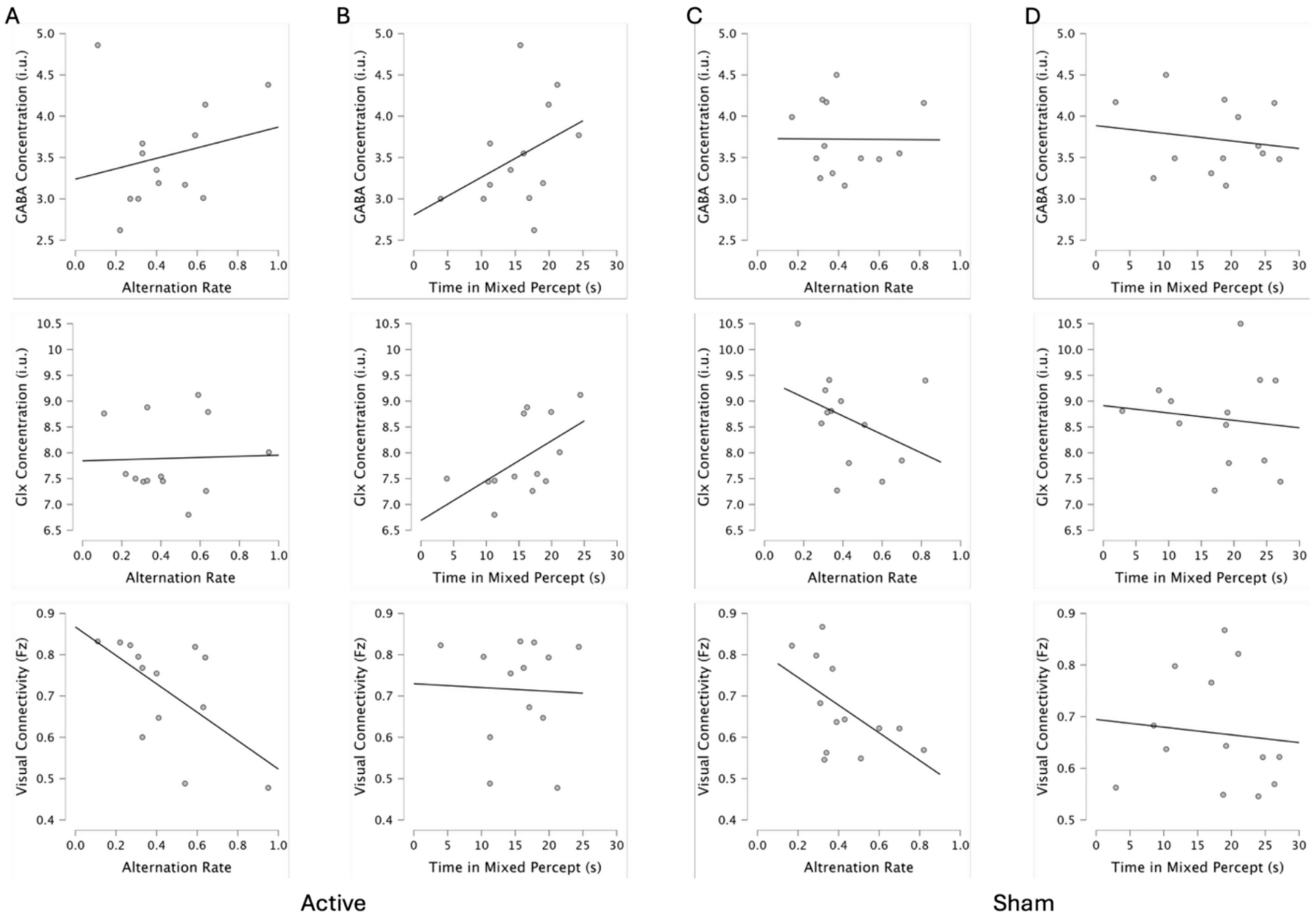
Correlation plots for active and sham conditions alternations rates **(A,C)** and time spent viewing mixed percept **(B,D)** with GABA concentration, Glx concentration, and visual connectivity.

Associations among GABA or Glx concentrations, binocular rivalry metrics, and visual cortex functional connectivity are shown in Table 1. We did not correct for multiple comparisons but rather assessed whether any correlation occurred at baseline for both the active and sham a-tDCS sessions to evaluate whether such a correlation was replicated across sessions. Binocular rivalry alternation rates were not correlated with GABA or Glx concentrations. There was a positive correlation between baseline visual cortex Glx concentrations and time spent viewing mixed percept in the active baseline condition (*p* = 0.46; Figure 4), but this was not replicated in the baseline condition prior to sham stimulation and therefore may have been a type 1 error. We found opposite correlations between binocular rivalry alternation rates and baseline visual cortex functional connectivity for the two conditions. There were no interactions between baseline GABA or Glx concentration and baseline visual cortex functional connectivity (*p* > 0.05).

**Table 1:** Associations among GABA or Glx concentrations, binocular rivalry metrics, and visual cortex functional connectivity for active and sham conditions before stimulation at baseline.

Baseline session	Correlation	Pearson’s r	*p*
Active	GABA * Alternation Rates	0.221	0.469
	GABA * Time Spent in Mixed Percept	0.386	0.193
	Glx * Alternation Rates	0.033	0.914
	Glx * Time Spent in Mixed Percept	0.561	0.046 *
	GABA * Visual Connectivity	−0.074	0.809
	Glx * Visual Connectivity	0.487	0.091
	Alternation Rates * Visual Connectivity	−0.605	0.029 *
	Time Spent in Mixed Percept * Visual Connectivity	−0.039	0.899
Sham	GABA * Alternation Rates	−0.007	0.981
	GABA * Time Spent in Mixed Percept	−0.160	0.602
	Glx * Alternation Rates	−0.360	0.226
	Glx * Time Spent in Mixed Percept	−0.118	0.702
	GABA * Visual connectivity	0.027	0.930
	Glx * Visual connectivity	0.088	0.774
	Alternation Rates * Visual Connectivity	0.553	0.050
	Time Spent in Mixed Percept * Visual Connectivity	−0.100	0.744

*p < 0.05.

## Discussion

We did not observe an effect of visual cortex a-tDCS on GABA concentration. This result contrasts with reports of reduced GABA concentration following motor cortex a-tDCS using similar experimental designs and sample sizes. Therefore, a-tDCS may have a different mechanism of action when delivered to the visual versus motor cortex. This is consistent with Castrillon et al. (2020) who found that repetitive transcranial magnetic stimulation—a different form of non-invasive brain stimulation – decreased inhibition in the frontal cortex, but increased inhibition in the occipital cortex. The current results are also consistent with our previous observation that visual cortex a-tDCS had no influence binocular rivalry dynamics (Abuleil et al., 2021).

Although the Glx concentration post active a-tDCS did not differ from post sham a-tDCS, pre-planned paired samples t-tests revealed a significant increase in Glx following active but not sham a-tDCS. It is possible that our study was underpowered to detect the interaction between Condition and Time, particularly as we found higher values for Glx prior to sham treatment compared with those prior to active treatment, which can only be attributed to random variation. Further investigation is indicated to substantiate an increase in visual cortex Glx concentration following active a-tDCS; such an effect would be consistent with increased visual cortex excitability following a-tDCS (Ding Z, et al., 2016).

In contrast to studies of other cortical areas, we found no effect of a-tDCS on resting-state functional connectivity in the visual cortex. Studies have showed increased functional connectivity following anodal tDCS to the motor cortex (Amadi et al., 2015; Bachtiar et al., 2015; Stagg et al., 2014) as well as the inferior frontal gyrus (Meinzer et al., 2012). One study of the sensorimotor network demonstrated a decrease in resting-state functional coupling following a-tDCS compared to the sham condition (Antonenko et al., 2017). A-tDCS may act differently when applied to the visual cortex (Cohan et al., 2023).

We did not replicate the association between visual cortex GABA concentration and binocular rivalry dynamics that has previously been reported (Mentch et al., 2019; van Loon et al., 2013). The relationship between GABA and visual perception is likely to be complex. For example, another study also failed to replicate the association between visual cortex GABA concentration and binocular rivalry dynamics in young adults, but the effect emerged when data from older and young adults were combined. Further, cortical GABA concentrations and binocular rivalry dynamics vary considerably from day to day both across and within participants, with influential factors such as alcohol intake (Donnelly and Miller, 1995), caffeine intake (George, 1936), menstrual cycle fluctuations (Epperson et al., 2005), and certain medications (Vetencourt et al., 2008). Nonetheless, we controlled for many of these factors except menstrual cycle with our inclusion criteria (e.g., use of psychoactive drugs) and provided instructions to participants (e.g., avoid alcohol the night before and caffeine the day of scanning). However, we could not objectively assess these potential confounds for the relationship between visual cortex GABA concentration and binocular rivalry dynamics.

It is important to note the limitations of this study. Although our results were non-significant, a true null effect cannot be confirmed with a small sample size, and further investigation is required. Recent evidence points to the variability of tDCS effects across participants and patients (Falcone et al., 2018) highlighting the potential importance of larger sample sizes. The interaction between condition and time was not significant, but a trend for an increase in glutamate concentration following real anodal tDCS may inform future studies. Additionally, the baseline measures for active and sham conditions were significantly different. There is no clear explanation for this aside from random variation. Our study also did not include electroencephalography (EEG) which some studies have used to assess the effects of tDCS (Ding Z, et al., 2016; Dong et al., 2020; Lau et al., 2021; Nakazono et al., 2020). This was due to physical limitations of the MRI space we used. The lack of VEP recordings limits our conclusions and analysis of the results.

Some studies of tDCS effects on the visual cortex have incorporated task-related neurophysiological measures to explore a more direct link between neural activity and visual perception and to assess active visual network engagement (Ahn et al., 2023; Callan et al., 2016; Spiegel et al., 2013a). Ahn et al. (2023) recently found that visual cortex a-tDCS increases the blood-oxygenation-level-dependent (BOLD) responses to dynamic visual patterns measured with fMRI. This highlights that task related neural activity is required to reveal certain visual cortex tDCS effects. In our study design, which focussed primarily on MRS measures, we aimed to replicate previous studies that did not include fMRI-tasks (Kim et al., 2014). However, the lack of an active task may have meant that we missed tDCS effects that are only evident when observing task-related neural activity within the visual cortex. Further exploration of this possibility is required. A recent systematic review has provided an overview of how to best combine tdcs and fMRI, and the benefits of doing so (Esmaeilpour et al., 2020), which include using fMRI for functional localization of visual areas or other areas of interest, as well as quantifying factors that have been found to predict tDCS responsiveness across patients.

It is possible that a-tDCS simply does not affect the visual cortex (Brückner and Kammer, 2016) since neither GABA concentration nor functional connectivity were modulated. However, visual cortex a-tDCS has been associated with behavioral changes such as modified visual perceptual learning (Peters et al., 2013), improved contrast sensitivity (Behrens et al., 2017; Ding Z, et al., 2016; Kraft et al., 2010; Spiegel et al., 2013a), enhanced perception and memory of faces and objects (Barbieri et al., 2016), and enhanced recovery of stereopsis in adults with amblyopia (Spiegel et al., 2013b). In addition, a recent meta-analysis identified reliable effects of visual cortex non-invasive brain stimulation on crowding and contrast sensitivity (Bello et al., 2023). Further exploration is required to identify the mechanisms underlying these effects.

## Conclusion

Visual cortex a-tDCS did not alter GABA concentration or resting-state functional connectivity. There was preliminary evidence of an increase in Glx following active a-tDCS. Mounting evidence implies that visual cortex compared to motor cortex is differentially affected by simulation (Cohan et al., 2023; Stoby et al., 2022). Our results also suggest that visual cortex a-tDCS may have a different mechanism of action than motor cortex a-tDCS.

## Data Availability

The raw data supporting the conclusions of this article will be made available by the authors, without undue reservation.

## Footnotes

^1^ http://www.fil.ion.ucl.ac.uk/spm

^2^ http://afni.nimh.nih.gov/afni

^3^ http://surfer.nmr.mgh.harvard.edu/
