# Supplementary material for: Anodal transcranial direct current stimulation does not alter GABA concentration or functional connectivity in the normal visual cortex
Source: Front Neurosci. 2025 Oct 15;19:1639838. doi: 10.3389/fnins.2025.1639838 (PMC12570334; doi:10.3389/fnins.2025.1639838)
Supplement: Supplementary file 1 [file Table_1.pdf]

Supplementary Table 1

Table 1: Mean ± SD of GABA and Glx concentrations in i.u. for N=13 participants.

| | **Visual Cortex GABA** | | **Visual Cortex Glx** | |
|---|---|---|---|---|
| | **Pre** | **Post** | **Pre** | **Post** |
| **Active** | 3.516 ± 0.177 | 3.378 ± 0.247 | 7.892 ± 0.206 | 8.495 ± 0.19 |
| **Sham** | 3.722 ± 0.119 | 3.734 ± 0.139 | 8.66 ± 0.251 | 8.795 ± 0.314 |
